# UPLC-MS/MS Method for Simultaneous Determination of Three Major Metabolites of Mequindox in Holothurian

**DOI:** 10.1155/2018/2768047

**Published:** 2018-04-01

**Authors:** Huihui Liu, Chuanbo Ren, Dianfeng Han, Hui Huang, Rongjie Zou, Huawei Zhang, Yingjiang Xu, Xianghong Gong, Xiuzhen Zhang, Yanshen Li

**Affiliations:** ^1^Shandong Marine Resource and Environment Research Institute, Laboratory of Restoration for Marine Ecology, Yantai 264006, China; ^2^College of Life Science, Yantai University, Yantai 264005, China

## Abstract

This study developed an ultraperformance liquid chromatography tandem mass spectrometry (UPLC-MS/MS) method for the detection of three major metabolites of mequindox, including 3-methyl-quinoxaline-2-carboxylic acid, 1-desoxymequindox, and 1,4-bisdesoxymequindox (MQCA, 1-DMEQ, and BDMEQ), in holothurian. Target analytes were simplified with ultrasound-assisted acidolysis extracted without complicated enzymolysis steps. After that, each sample was centrifuged and purified by an Oasis MAX cartridge. Then, the processed samples were separated and monitored by UPLC-MS/MS. This developed method has been validated according to FDA criteria. At fortified levels of 2, 10, and 20 *μ*g/kg, recoveries ranged from 82.5% to 93.5% with the intraday RSD less than 7.27% and interday RSD less than 11.8%. The limit of detection (LOD) of all the three metabolites ranged from 0.21 to 0.48 *μ*g/kg, while the limit of quantification (LOQ) ranged from 0.79 to 1.59 *μ*g/kg. On application to commercial samples, 14 of 20 samples were detected positive for the three target analytes, with positive rate at 70 percentage. The result indicated that this method was specific, sensitive, and suitable for the quantification and conformation of the three major metabolites of MEQ in holothurian.

## 1. Introduction

Mequindox (MEQ, 3-methyl-2-quinoxaline acetyl-1,4-dioxide) is a newly synthetic quinoxaline-1,4-dioxides (QdNOs) antibiotic. The QdNOs have been widely applied as growth-promoting agents in animal husbandry in last decades [1, 2]. Recently, due to the high-antimicrobial activity, QdNOs are also used in aquaculture, especially in holothurian culture [3]. However, recently literatures showed that the toxicity of QdNOs is high, which might induce potential carcinogenicity and mutagenicity [4–6]. It was also reported that MEQ might also induce liver and adrenal toxicity at high or low dose after oral administration [7, 8]. MEQ was also reported to cause methylation of holothurian DNA, which was one of the expression forms of DNA damage [9, 10]. Now, as members of QdNOs, carbadox and olaquindox (CBX and OLA) have been banned as the feed additives in the past decades [4–6]. Therefore, MEQ and its metabolites residues should be paid close attention. What is more, recent metabolism investigations reported that the metabolites of QdNOs might also induce severe toxicity to animals and humans [11, 12].

Many metabolites of MEQ were detected, and structure was identified in previous metabolic investigations [13–16]. Specially, the major metabolic pathways were N→O group reduction and carboxylation [17]. In these reports, the major metabolites were 1-desoxymequindox (1-DMEQ) and 1,4-bisdesoxymequindox (BDMEQ) from N→O group reduction. As another metabolite, 3-quinoxaline-2-carboxylic acid (MQCA) was also detected as a metabolic product of carboxylation. After administration, MEQ might be metabolized to different metabolites in holothurian. Considering the potential high toxicity of MEQ, it should also be paid close attention to control the residues in foods.

For the control of these chemical compounds in foods, there were many literatures reported for the detection of QdNOs and the metabolites in the previous studies, such as high-performance liquid chromatography (HPLC) coupled with ultraviolet (UV) detector [18–20] and high-performance liquid chromatography-tandem mass spectrometry (LC-MS/MS) [21–24]. Mass spectrometry is now widely applied for QdNOs and metabolites detection in foods due to the high sensitivity and selectivity and allows spread range for target analytes.

**Figure 1:**
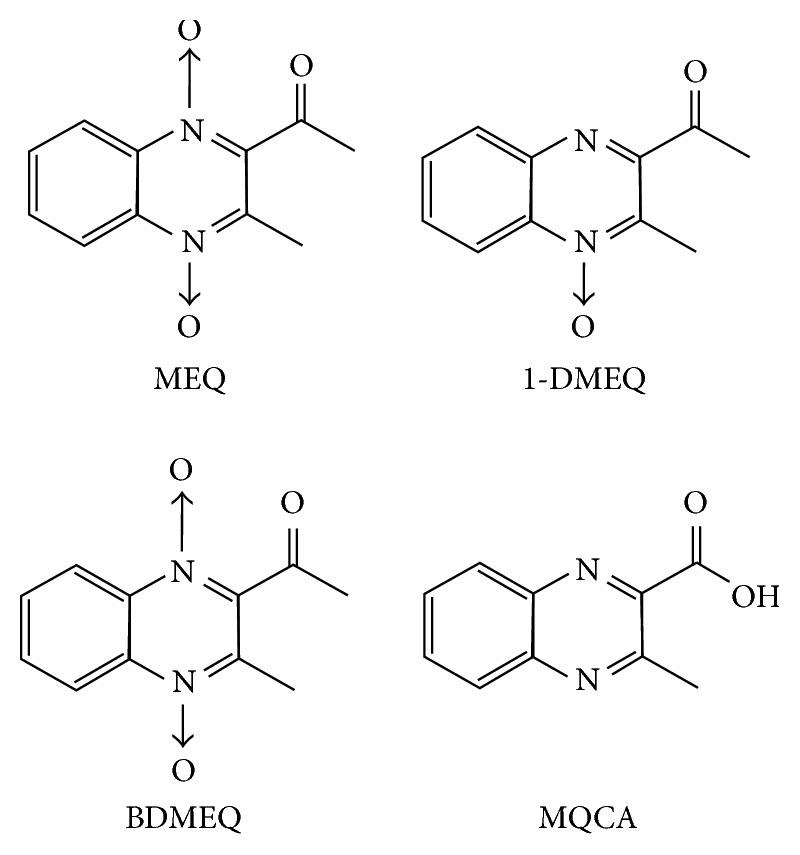
Chemical structures of 1-DMEQ, BDMEQ, MEQ, and MQCA.

However, these current methods mainly focused on the land animal-derived foods. To the best of our knowledge, there were no related methods for the detection of MEQ and metabolites in aquatic animal-derived foods, especially in holothurian. In this work, for the control of MEQ residues in holothurian, we presented a novel, sensitive, and rapid UPLC-MS/MS (ultraperformance liquid chromatography coupled with triple-quadrupole mass spectrometry) protocol for simultaneous detection of the three major metabolites of MEQ (MQCA, 1-DMEQ, and BDMEQ structures are shown in Figure 1) in holothurian. The extraction procedure was processed with ultrasound assistance, and target analytes were extracted using hydrochloric acid without complicated enzymolysis steps. Until now, there are no marker residue metabolites of MEQ in holothurian. This work will contribute to the control of MEQ in aquatic animal-derived foods, and it will also be benefit for further pharmacokinetics investigations in holothurian.

## 2. Materials and Methods

### 2.1. Reagents and Chemicals

MQCA (98.0%) was purchased from Dr. Ehrenstorfer GmbH (Augsburg, Germany). 1-desoxymequindox (1-DMEQ, 98.0%) and bisdesoxymequindox (BDMEQ, 98.0%) were synthesized by Green Bayer Biotechnology Company (Wuhan, China).

HPLC grade acetonitrile was purchased from Dima Technology Inc. (Muskegon, MI, USA). HPLC grade formic acid was obtained from Fisher Scientific Inc. (Pittsburgh, PA, USA). The Milli-Q synthesis system was adopted for the production of deionized water (Millipore, MA, USA). Oasis MAX SPE cartridges (3 cc, 60 mg) were purchased from Agilent Technologies (Santa Rosa, CA, USA). Other reagents and chemicals used in this work were of analytical grade which were purchased from the Sinopharm Chemical Reagent Co., Ltd. (Beijing, China).

Stock standard solutions of MQCA (1.0 mg/mL), 1-DMEQ (1.0 mg/mL), and BDMEQ (1.0 mg/mL) were prepared by dissolving 5 mg of each standard in 5 mL of methanol. These stock standard solutions were stored at −20°C in brown amber bottles until use, and they were stable for at least 3 months. Working mixed standard solutions were prepared by diluting the stock standard solutions with methanol. These working solutions were stored at −20°C in the brown amber bottle and stable for at most 1 week.

### 2.2. Apparatus

A model HQ-60 vortex mixer was purchased from North TZ-Biotech Development Co. (Beijing, China). Nitrogen evaporation equipped with an Evap 111 evaporator was purchased from Organomation Associates Inc. (Berlin, MA, USA). Tissue homogenizer was purchased from MeiDi (Foshan, China). Syringe filters (0.22 *μ*m) were obtained from Pall Corp. (Ann Arbor, MI, USA). Centrifuge (5804 R) was obtained from Eppendorf (Hamburg, Germany).

The mixed targets of the three metabolites were were separated via an Acquity BEH C18 column (100 mm × 2.1 mm i.d., 1.7 μm particle size) on a Waters AcquityTM UPLC system (Waters, Milford, MA, USA). The column oven temperature was maintained at 40°C. The mobile phase was composed of solvent A (acetonitrile containing 0.5% formic acid) and solvent B (water containing 0.5% formic acid). Gradient elution was adopted in this research, and the program was performed as follows: 0–0.25 min, 5% A; 0.25–7.75 min, 5–95% A; 7.75–8.50 min, maintain 95% A; 8.50–8.51 min, 95–5% A; and 8.51–10.0 min, maintain 5% A. The flow rate was settled at 0.25 mL/min with an injection volume of 10 *μ*L. 10% acetonitrile in water and 100% acetonitrile were used as weak and strong wash solvents, respectively.

The Mass Quattro Premier XE triple quadrupole mass spectrometer (Waters, Manchester, UK) was fitted to UPLC system with an electrospray ionization (ESI) source. Typical source conditions were optimized for maximum intensity as follows: desolvation gas at 700 L/h with the temperature of 350°C, capillary voltage at 3.0 kV, source temperature at 80°C, cone gas flow rate at 50 L·h^−1^, and collision at 0.11 mL/min. For all compounds, MS instrument was operated in ESI positive (ESI+) multiple reaction monitoring (MRM) mode. Optimized MS/MS parameters are summarized in Table 1.

### 2.3. Sample Preparation

Blank holothurian samples were obtained from the breeding base of Shandong Marine Resource and Environment Research Institute (Yantai, Shandong, China) which were previously characterized using UPLC-MS/MS. Samples were homogenized by a domestic food blender and then stored at −20°C till use.

An amount of 5.00 g (±0.025 g) of holothurian samples was weighed and transferred into a 50 mL polypropylene centrifuge tube. Samples were divided into four groups. Three fortified groups were prepared by adding mixed working standard solution to yield final concentrations of 2, 10, and 20 *µ*g/kg each. One unfortified group was set as the negative control by adding methanol at equal volume only. After fortification, each sample was vortexed for 30 s and settled at room temperature in a dark place for 20 min for incubation. Then, the extraction solvent of 15 mL hydrochloric acid (2 mol/L) was added, and each sample was homogenized for 30 s. Ultrasound-assisted extraction for 60 min was processed in this work for samples. After that, samples were centrifuged at 10,000 g for 10 min at 4°C, and the supernatant was transferred to another 50 mL tube for the next purification.

Oasis MAX SPE cartridges (3 cc, 60 mg, Waters, USA), which were mainly consisted of anionite, were adopted for the purification process. The cartridges were conditioned with 3 mL of methanol and 3 mL of hydrochloric acid (2 mol/L). Then, the supernatant was loaded onto the prepared cartridge by gravity. Each was rinsed with 3 mL of sodium acetate-methanol solution (0.05 mol/L, 6.12 g of sodium acetate was dissolved in 900 mL of water and adjusted to pH 7.0 with sodium hydroxide, and then, the volume was kept constant at 1000 mL). Before elution, each cartridge was dried with a vacuum pump, and then target analytes were eluted with 3 mL of methanol/ethyl acetate (2/98, *v/v*). The elution was dried with anhydrous sodium sulfate, and then further dried under a gentle flow of nitrogen at 40°C. The residues were redissolved with acetonitrile with 0.1% formic acid and filtered through a 0.22 *μ*m syringe filter into an autosampler vial for further UPLC-MS/MS analysis.

### 2.4. Method Validation

The linearity, limit of detection (LOD), limit of quantification (LOQ), and accuracy and precision were validated for this method according to the FDA criteria [25].

#### 2.4.1. Linearity

The linearity was validated by matrix-matched calibration curves by preparing external standard calibrations. Matrix-matched standards were prepared at 8 point concentrations of 1, 2, 5, 10, 20, 50, 100, and 200 ng/mL by adding working mixed standard solutions to negative control samples.

#### 2.4.2. LOD and LOQ

LOD is determined by a signal-to-noise ratio (S/N) ≥ 3, and it stands for the lowest concentration for the detection of each analyte. LOQ is determined by S/N ≥ 10, and it stands for the lowest measured concentration of each analyte.

#### 2.4.3. Accuracy and Precision

Each analyte in spiked samples was detected in this developed procedure with six replicates on three separate days with the spiked levels at 2, 10, and 20 *μ*g/kg. Concentration of each sample was calculated according to the calibration curves. The recovery was determined by the peak area of measured concentration compared with the standard concentration. Accuracy and precision were evaluated by determining recoveries and intraday and interday relative standard deviation (RSD).

## 3. Results and Discussion

### 3.1. Optimization of Extraction Procedure

In order to obtain a satisfactory recovery, the first and most crucial step is the extraction procedure. In the previous literature, ethyl acetate was evaluated for the extraction of these target analytes in animal tissues [20, 26, 27]. Moreover, the extraction of the compounds from tissues requires complicated acidolysis, alkaline hydrolysis, or enzymolysis [3].

In order to simplify the extraction process, in this work, ethyl acetate and hydrochloric acid (2 mol/L) were evaluated as the extraction solution without acidolysis, alkaline hydrolysis, or enzymolysis procedure. The results showed that ethyl acetate led to a low recovery for MQCA, which was in accordance with the previous studies [1, 27]. However, hydrochloric acid (2 mol/L) performed as the extraction solution could lead to the satisfactory result. The acid solution could dissociate the binding metabolites and lead to a higher recovery over 90% without complicated enzymolysis procedure. Therefore, hydrochloric acid (2 mol/L) was adopted as the extraction solution.

The ultrasound-assisted extraction time was also evaluated to obtain higher recovery in this research at 15, 30, 45, 60, 90, and 120 min. The results showed that the recoveries for each metabolite increased with the ultrasound time during 60 min. Specially, when the extraction reached 60 min, the recovery for each metabolite was satisfactory, and 90 and 120 min extraction time did not lead to significant higher recoveries. Therefore, 60 min ultrasound-assistedextraction time was adopted in this research.

### 3.2. Optimization of Cleanup Procedure

For trace amount analysis, it is important to get rid of the matrix inhibition in order to enhance the sensitivity in UPLC-MS/MS analysis process. Based on the previous literatures, the most common SPE cartridges for the purification of MEQ metabolites were Bond Elut C18, HLB, MAX, and MCX to eliminate possible interferences from crude sample extract [19, 21]. MAX and MCX cartridges are associated with pKa value of analytes and pH value of loading solution. HLB cartridge depends on the intermolecular forces between analytes and the packing.

In this research, MAX, MCX, and HLB cartridges were evaluated for the purification of target analytes. The result is shown in Table 2. From the table, MCX was not suitable due to the extremely low recoveries for all the analytes. HLB cartridge was not suitable either due to the unsatisfactory recoveries no more than 50% for each analytes. As to MAX cartridge, satisfactory recoveries of all the analytes were obtained, which was in accordance with the previous literatures [19, 23]. Therefore, MAX cartridges were adopted for the purification process.

### 3.3. UPLC-MS/MS Analysis

UPLC was performed in this research via an Acquity BEH C18 column. The separation condition was optimized with different mobile phases, percentage of formic acid, and gradient elution program. These target analytes could be separated within 4 min using a micromass Quattro Premier XE triple quadrupole mass spectrometer coupled to UPLC system by fitting with electrospray ionization (ESI). All the analytes exhibited high response in the positive mode (ESI^+^). Acetonitrile (containing 0.5% formic acid) and water (containing 0.5% formic acid) were adopted as the mobile phase in order to improve the ionization efficiency of mass spectrometer detection.

The MRM conditions of each analytes were optimized by injecting standard solutions. The most optimized parameters adopted in this research are summarized in Table 1. This developed MS/MS method fulfills the EU's technical criteria [28] with four identification points (IPs).

### 3.4. Method Validation

**Figure 2:**
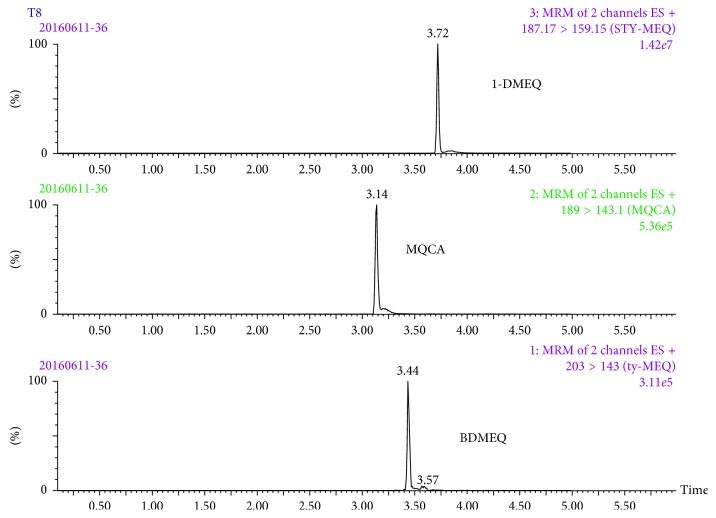
MRM chromatograms of blank holothurian samples fortified at 10 *µ*g/kg.

Negative holothurian samples were analyzed to verify the specificity of the proposed analytical method. MRM chromatograms of fortified samples spiked at 10.0 *μ*g/kg are shown in Figure 2. From the figure, it can be observed that there were no interferences around the retention time of each analyte.

The linearity was evaluated, and matrix-matched linear regression calibration curves ranged from 1.0 to 200 ng/mL are shown in Table 3. The equations were *y* = 365.31*x* + 1329.4 for 1-DMEQ, *y* = 621.55*x* + 335.41 for BDMEQ, and *y* = 547.98*x* + 157.67 for MQCA. The correlation coeffcients (*r*^2^) for each analyte were over 0.99.

LODs determined by S/N ≥ 3 in this research ranged from 0.21 to 0.48 *μ*g/kg, and LOQs determined by S/N ≥ 10 ranged from 0.79 to 1.59 *μ*g/kg for each analyte in holothurian samples, respectively (Table 3).

Accuracy and precision were determined by the recoveries of each analyte in fortified sample. Each analyte in the spiked levels was analyzed with six replicates each day (*n* = 6) on three separate days. Mean recoveries for all the analytes ranged from 82.5% to 93.5% with the intraday RSD less than 7.27% and interday RSD less than 11.8% (Table 4).

### 3.5. Application to Real Samples

The developed analytical method was successfully applied for the determination of all the 3 metabolites in 20 samples obtained from the local supermarket. Totally, 14 positive samples were detected positive with concentrations of 1-DMEQ, BDMEQ, and MQCA ranged from 2.27 to 6.44 *μ*g/kg, 3.69 to 6.88 *μ*g/kg, and 3.57 to 9.22 *μ*g/kg, respectively (Table 5).

## 4. Conclusion

This research describes a novel ultrasound-assisted acidolysis-based UPLC-MS/MS method for the determination of three major metabolites of mequindox (1-DMEQ, BDMEQ, and MQCA) in holothurian samples. Target analytes were simplified with ultrasound-assisted acidolysis extracted without complicated enzymolysis steps. After that, each sample was centrifuged and purified by an Oasis MAX cartridge. Then, the processed samples were separated and monitored by UPLC-MS/MS. At fortified levels, the developed method exhibited satisfactory performance with good linearity, lower LODs, and good accuracy and precision. On application to commercial samples, 14 of 20 samples were detected positive for the three target analytes, with positive rate at 70 percentage. The result indicated that this method was specific, sensitive, and suitable for the quantification and conformation of the three major metabolites of MEQ in holothurian.

## Figures and Tables

**Table 1:** MS/MS parameters for MQCA, 1-DMEQ, and BDMEQ in the positive electrospray ionization mode.

Compound	Parent ion (*m*/*z*)	Daughter ion (*m*/*z*)	Cone voltage (V)	Collision energy (eV)
1-DMEQ	203	143^∗^	30	25
		185		17
BDMEQ	187.1	145.1	33	25
		159.15^∗^		20
MQCA	189	92	19	24
		143.1^∗^		17

^∗^Quantitative ion.

**Table 2:** SPE purification efficiency of MCX, MAX, and HLB cartridges for target analytes.

SPE cartridge	Recovery (%)		
	1-DMEQ	BDMEQ	MQCA
MCX	3.21%	4.23%	2.38%
MAX	88.31%	92.58%	99.63%
HLB	42.38%	36.54%	38.82%

**Table 3:** Parameters (including the standard curve, LOD, and LOQ) of MQCA, 1-DMEQ, and BDMEQ in holothurian samples.

Matrix	Analyte	Linear range (ng/mL)	Regression equitation	*r* ^2^	LOD (*µ*g/kg)	LOQ (*µ*g/kg)
Chicken	1-DMEQ	1.0∼200	*y* = 365.31*x* + 1329.4	0.9982	0.48	1.59
	BDMEQ	1.0∼200	*y* = 621.55*x* + 335.41	0.9996	0.21	0.69
	MQCA	1.0∼200	*y* = 547.98*x* + 157.67	0.9995	0.24	0.79

**Table 4:** The accuracy and precision for the analysis of MQCA, 1-DMEQ, and BDMEQ residues in holothurian.

Matrix	Analyte	Spiked level (*µ*g/kg)	Mean recovery (%)	Intraday RSD% (*n* = 6)	Interday RSD% (*n* = 18)
Holothurian	1-DMEQ	2	88.0	4.96	11.8
		10	83.7	6.07	6.27
		20	90.5	6.96	8.06
	BDMEQ	2	88.0	3.14	5.85
		10	82.5	5.90	6.51
		20	88.5	7.27	7.95
	MQCA	2	93.5	3.82	5.37
		10	85.7	5.56	6.94
		20	85.5	3.80	6.61

**Table 5:** Concentrations of 1-DMEQ, BDMEQ, and MQCA contamination in commercial holothurian samples.

Commercial sample	Sample code	Concentration of 1-DMEQ (*μ*g/kg)	Concentration of BDMEQ (*μ*g/kg)	Concentration of MQCA (*μ*g/kg)
Holothurian	1	ND	ND	ND
	2	2.27	4.68	3.99
	3	ND	6.88	6.52
	4	3.96	6.24	7.96
	5	5.68	3.95	ND
	6	6.26	6.21	ND
	7	6.44	3.698	ND
	8	ND	ND	ND
	9	ND	ND	ND
	10	3.98	5.68	3.57
	11	ND	ND	ND
	12	ND	ND	9.22
	13	4.69	6.09	8.69
	14	ND	ND	ND
	15	6.10	4.33	ND
	16	ND	ND	5.39
	17	ND	ND	ND
	18	5.62	4.18	ND
	19	5.33	5.62	3.59
	20	ND	5.19	ND

ND: not detected.
